# A retrospective study comparing the severe cutaneous adverse reactions in patients receiving triazole antifungal therapy and literature analysis

**DOI:** 10.1093/mmy/myag084

**Published:** 2026-08-17

**Authors:** Cheng-Tao Jin, Qian-Qian Jiang, Ping Chen, Wei Jiang

**Affiliations:** Department of Pharmacy, Sir Run Run Shaw Hospital, Zhejiang University School of Medicine, Hangzhou 310016, China; Department of Pharmacy, Traditional Chinese Medicine Hospital of Changshan, Quzhou 324200, China; Department of Pharmacy, The Quzhou Affiliated Hospital of Wenzhou Medical University, Quzhou People’s Hospital, Quzhou 324000, China; Department of Pharmacy, The Quzhou Affiliated Hospital of Wenzhou Medical University, Quzhou People’s Hospital, Quzhou 324000, China

**Keywords:** triazole antifungal drugs, severe cutaneous adverse reactions, time-to-onset, FDA Adverse Event Reporting System, signal strength

## Abstract

Triazole antifungal drugs (TADs) are widely used in clinical practice but carry a risk of severe cutaneous adverse reactions (SCARs). However, comprehensive real-world evidence regarding the comparative safety profiles of individual TADs remains limited. This research aimed to investigate and compare the real-world risk, clinical characteristics, and time-to-onset of SCARs associated with individual TADs. The SCAR cases with TADs as the primary suspect drug were identified in the FDA Adverse Event Reporting System. Disproportionality analysis and Bayesian methods were employed to detect safety signals with Reporting Odds Ratio (ROR), Proportional Reporting Ratio (PRR), Bayesian Confidence Propagation Neural Network (BCPNN), and Empirical Bayes Geometric Mean (EBGM). Differences of clinical features, outcomes, time-to-onset (TTO), and signal strength at the preferred term (PT) level were analyzed. A total of 1429 SCAR cases with five TADs were identified. Fluconazole showed the strongest and most consistent statistical association across all four algorithms (ROR = 5.2), followed by itraconazole. Voriconazole yielded mixed signals (positive by ROR and BCPNN only), while Posaconazole and Isavuconazole exhibited no significant SCAR signals. Voriconazole was associated with the highest mortality proportion (12.8%), followed by posaconazole (10%). The median TTO for SCARs was 8 days, with voriconazole having the longest delay (16 days). At the PT level, 45 distinct signals were identified, including high-risk manifestations such as fixed eruption (ROR = 24.43), mucosal ulceration, and SJS/TEN overlap. This study identified significant variability of SCARs signals among triazole antifungals. These findings support early risk recognition and informed antifungal selection in clinical practice.

## Introduction

Severe cutaneous adverse reactions (SCARs) are a group of critical dermatological conditions that can be triggered by various factors, including medications, infections, and underlying systemic diseases. These reactions encompass Acute Generalized Exanthematous Pustulosis (AGEP), Stevens-Johnson Syndrome (SJS), Toxic Epidermal Necrolysis (TEN), Drug Reaction with Eosinophilia and Systemic Symptoms (DRESS), as well as Erythema Multiforme (EM). These conditions may lead to multi-organ failure, increased mortality risk, and prolonged hospitalization.^1–4^ Based on the extent of body surface area (BSA) detachment, the SJS/TEN spectrum is classified into three major categories: SJS (<10% BSA detachment), SJS/TEN overlap (10%–30% BSA detachment), and TEN (>30% BSA detachment).^5,6^ These criteria apply specifically to the Stevens–Johnson syndrome and toxic epidermal necrolysis spectrum, whereas other SCAR subtypes such as AGEP and DRESS have distinct diagnostic criteria.

The triazole antifungal drugs (TADs), encompassing fluconazole, itraconazole, voriconazole, posaconazole, and isavuconazole, are broad-spectrum antifungal agents in the prevention and treatment of invasive fungal diseases primarily stemming from immunodeficiency.^7,8^ According to relevant guidelines, TADs are recommended as the preferred therapy for the management of candidemia and non-neutropenic invasive pulmonary aspergillosis, as well as for the prevention of invasive candidiasis in ICU settings. This recommendation aligns with the updated guidelines on aspergillosis from the Infectious Diseases Society of America (IDSA), revised in 2016.^9,10^ The most commonly reported side effects of TADs include dryness, drug eruptions, alopecia, diarrhea, fatigue, nausea, and vomiting. Additionally, they may also lead to QT interval prolongation.^11–17^ Moreover, TADs frequently affect the skin and its appendages. For instance, the package insert for voriconazole injection mentions that it can induce SJS, TEN, and EM. However, there is currently a limited number of studies on TAD-induced SCARs, with a preponderance of case reports but a scarcity of real-world research. Therefore, it is essential to comprehensively and in-depth describe SCARs caused by TADs.

The data used in this study were sourced from the FDA Adverse Event (AE) Reporting System (FAERS), a database extensively employed in real-world safety research.^18–20^ Using the FAERS database, we retrieved reports on SCARs associated with TADs. We then applied advanced data-mining algorithms to analyze the correlation between TADs and SCARs, aiming to gain a more comprehensive understanding of their safety profile in clinical practice.

## Date and methods

### Data sources

Data from the first quarter of 2004 to the third quarter of 2024 were extracted from the FAERS database. A retrospective pharmacovigilance study was then conducted on SCARs associated with TADs. The FAERS database, which is released quarterly, comprises seven distinct subsets of data: patient demographic information (DEMO), drug information (DRUG), adverse event information (REAC), patient outcome information (OUTC), report source information (RPSR), drug therapy date information (THER), and drug indications (INDI).^21,22^

We processed the FAERS data by removing duplicate entries in accordance with FDA recommendations using R software.^23^ Specifically, when two or more reports shared the same CASEID (the identifier used for cases in the FAERS database), we retained the one with the most recent FDA_DT (the date on which the FDA received the case). In instances where both the CASEID and FDA_DT were identical, we selected the report with the higher PRIMARYID (the unique identifier for each FAERS report).^.23^ Adverse drug reactions and indications are coded at the preferred term (PT) level using the Medical Dictionary for Regulatory Activities (MedDRA, version 26.1). This version was consistently applied throughout the data extraction and analysis period (first quarter of 2004 to third quarter of 2024). Based on the association between the drug and the adverse drug reaction, drugs can be classified into three categories by analysts: ‘‘Primary Suspect’’ (PS), ‘‘Secondary Suspect’’ (SS), and ‘‘Concomitant’’ (C). The FAERS database is updated quarterly and is publicly accessible via the official website of the FDA. Additionally, the RPSR (Report Source) table aggregates reports voluntarily submitted from various sources, including patients, healthcare professionals (such as physicians, pharmacists, nurses, etc.), and manufacturers.

### Data extraction

**Figure 1.**
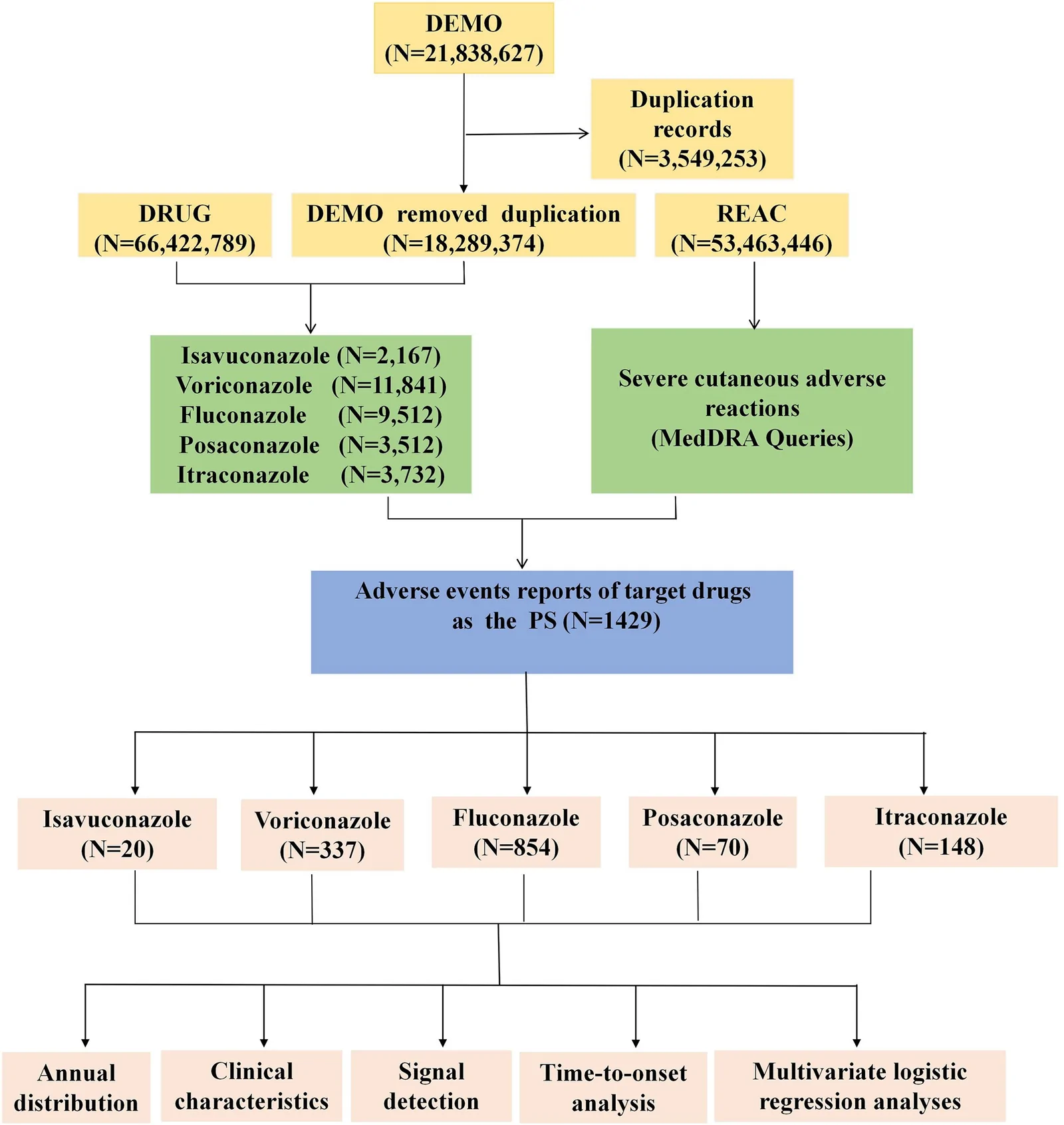
A flowchart illustrating the selection process for TADs-related Severe cutaneous adverse reactions from the FAERS database.

Through the FAERS database, this study extracted all reported cases involving TADs as the PS drugs. We obtained a total of 21 838 627 reported cases where TADs were identified as the PS drugs (Fig. 1). The core objective was to identify AEs associated with these specific medications, focusing on those linked to restricted target drugs with the generic names ‘‘Isavuconazole,’’ ‘‘Voriconazole,’’ ‘‘Fluconazole,’’ ‘‘Posaconazole,’’ or ‘‘Itraconazole’’ and their entry terms (https://www.ncbi.nlm.nih.gov/mesh/). SCARs were defined using the MedDRA PTs encompassing AGEP, SJS, TEN, DRESS, EM, fixed drug eruption, and other related severe cutaneous conditions as listed in the MedDRA hierarchy. Fixed drug eruption was included based on its classification as a SCAR in MedDRA, despite being generally less life-threatening than SJS/TEN, to ensure comprehensive capture of clinically significant dermatologic events. This study utilized regulatory activity-related terms from the Medical Dictionary for Drug Regulatory Activities (MedDRA) for analysis. The flowchart depicted in Figure 1 outlines the multi-step process encompassing data extraction, processing, and analysis.

### Data analysis

This study employed the disproportionality analysis method and Bayesian approaches, which are commonly utilized analytical tools in the field of pharmacovigilance. Disproportionality analysis compares the proportion of AEs reported for the target drug versus other drugs and incorporates metrics such as the Reporting Odds Ratio (ROR) and the Proportional Reporting Ratio (PRR).^.24^ Another Bayesian approach integrates two algorithms: the Bayesian Confidence Propagation Neural Network (BCPNN) and the Empirical Bayes Geometric Mean (EBGM) method.^.25^ The study employed these four distinct algorithms to enhance the reliability of the analysis regarding the association between drugs and adverse events. These algorithms were specifically utilized to quantify the correlation between TADs and SCARs. Drawing upon the four-cell table (Supplementary Table 1) from the disproportionality analysis method and incorporating Bayesian principles, the formulas and signal detection criteria for these four algorithms are elucidated in Supplementary Table 2.^22^

Generally speaking, an increase in the algorithm-derived values often indicates a stronger signal intensity, suggesting a potentially stronger association between the drug and the adverse event. In this study, time-to-onset (TTO) was defined as the interval between the AE date (EVENT_DT) and the TAD initiation date (START_DT). During the data preprocessing stage, all anomalous records with temporal inconsistencies (i.e., instances where EVENT_DT preceded START_DT) were systematically removed to ensure the temporal plausibility of the analyzed cases.^.26^ The median was used to describe the TTO of AEs. Building upon this, we further explored the impact of different stratification dimensions on the association between the target drugs and SCARs. Specifically, stratified analyses were conducted based on concomitant medication use (monotherapy vs. combination therapy) and reporter type (healthcare professionals vs. consumers).

### Statistical analysis

For every AE report, we extracted demographic variables, administrative descriptors, patient outcome, and the interval (in days) between treatment start and event onset. All data preprocessing, including deduplication, and statistical analyses were performed with R v4.2.3. (Vienna, Austria).

## Results

### Clinical characteristics

After removing duplicates from the FAERS database, we identified 1429 cases of SCARs associated with TADs, including Isavuconazole (20), Voriconazole (337), Fluconazole (854), Posaconazole (70), and Itraconazole (148) (Fig. 1). The clinical characteristics of the patients are presented in Table 1. Specifically, SCARs were more prevalent among males treated with Isavuconazole, Fluconazole, Posaconazole, and Itraconazole. Body weight was not consistently recorded for patients who developed SCARs during TAD therapy; however, among those with documented weights, SCARs predominantly occurred in patients weighing between 50 and 100 kg. The majority of SCARs cases occurred in patients aged 18–65 years, followed by those aged 66–85 years, with fewer cases reported in other age groups. Notably, severe outcomes included death, life-threatening conditions, disability, hospitalization or prolonged hospitalization, and other serious consequences. Among these, Posaconazole and Itraconazole were associated with a higher proportion of SCARs leading to hospitalization or prolonged hospitalization.

**Table 1.** Clinical characteristics of Triazole antifungal drug-related SCARs.

Characteristic	Isavuconazole	Voriconazole	Fluconazole	Posaconazole	Itraconazole
	(*N* = 20)	(*N* = 337)	(*N* = 854)	(*N* = 70)	(*N* = 148)
**Gender**					
Female	11 (55.0%)	156 (46.3%)	547 (64.1%)	46 (65.7%)	78 (52.7%)
Male	9 (45.0%)	159 (47.2%)	227 (26.6%)	23 (32.9%)	45 (30.4%)
Unknown	0 (0%)	22 (6.5%)	80 (9.4%)	1 (1.4%)	25 (16.9%)
**WT**					
<50 kg	0 (0%)	29 (8.6%)	33 (3.9%)	14 (20.0%)	4 (2.7%)
>100 kg	0 (0%)	13 (3.9%)	24 (2.8%)	0 (0%)	1 (0.7%)
50∼100 kg	2 (10.0%)	95 (28.2%)	257 (30.1%)	14 (20.0%)	23 (15.5%)
Unknown	18 (90.0%)	200 (59.3%)	540 (63.2%)	42 (60.0%)	120 (81.1%)
**Age/years**					
<18	1 (5.0%)	40 (11.9%)	16 (1.9%)	7 (10.0%)	13 (8.8%)
18∼65	10 (50.0%)	149 (44.2%)	513 (60.1%)	32 (45.7%)	82 (55.4%)
66∼85	4 (20.0%)	104 (30.9%)	173 (20.3%)	22 (31.4%)	16 (10.8%)
>85	0 (0%)	2 (0.6%)	18 (2.1%)	1 (1.4%)	1 (0.7%)
Unknown	5 (25.0%)	42 (12.5%)	134 (15.7%)	8 (11.4%)	36 (24.3%)
**Occupation of reporters**					
Consumer	16 (80.0%)	55 (16.3%)	165 (19.3%)	9 (12.9%)	9 (6.1%)
Health-Professional	0 (0%)	47 (13.9%)	70 (8.2%)	5 (7.1%)	32 (21.6%)
Lawyer	0 (0%)	0 (0%)	3 (0.4%)	0 (0%)	0 (0%)
Physician	1 (5.0%)	107 (31.8%)	291 (34.1%)	39 (55.7%)	73 (49.3%)
Other health-professional	2 (10.0%)	75 (22.3%)	196 (23.0%)	14 (20.0%)	21 (14.2%)
Pharmacist	1 (5.0%)	39 (11.6%)	76 (8.9%)	2 (2.9%)	12 (8.1%)
Registered Nurse	0 (0%)	0 (0%)	1 (0.1%)	0 (0%)	0 (0%)
Unknown	0 (0%)	14 (4.2%)	52 (6.1%)	1 (1.4%)	1 (0.7%)
**Outcomes**					
Death	1 (5.0%)	43 (12.8%)	66 (7.7%)	7 (10.0%)	5 (3.4%)
Disability	0 (0%)	2 (0.6%)	21 (2.5%)	0 (0%)	2 (1.4%)
Hospitalization-Initial or Prolonged	9 (45.0%)	89 (26.4%)	252 (29.5%)	33 (47.1%)	77 (52.0%)
Life-Threatening	2 (10.0%)	16 (4.7%)	53 (6.2%)	8 (11.4%)	5 (3.4%)
Other Serious (Important Medical Event)	5 (25.0%)	127 (37.7%)	377 (44.1%)	14 (20.0%)	54 (36.5%)
Required Intervention to Prevent Permanent Impairment	0 (0%)	2 (0.6%)	4 (0.5%)	0 (0%)	0 (0%)
Unknown	3 (15.0%)	58 (17.2%)	81 (9.5%)	8 (11.4%)	5 (3.4%)
**Reported countries (Top 3)**					
1	CH(40%)	US(31.8%)	US(26.2%)	FR(41.4%)	IND(29.1%)
2	US(25%)	FR(20.8%)	GB(20.4%)	US(20.0%)	CN(12.8%)
3	FR(10%)	JP(9.5%)	FR(17.4%)	CN(8.6%)	US(10.1%)

Abbreviations: US, United States; GB, United Kingdom; FR, France; CN, China; JP, Japan; CH, Switzerland; IND, India.

### The year of SCARs reports related to TADs

**Figure 2.**
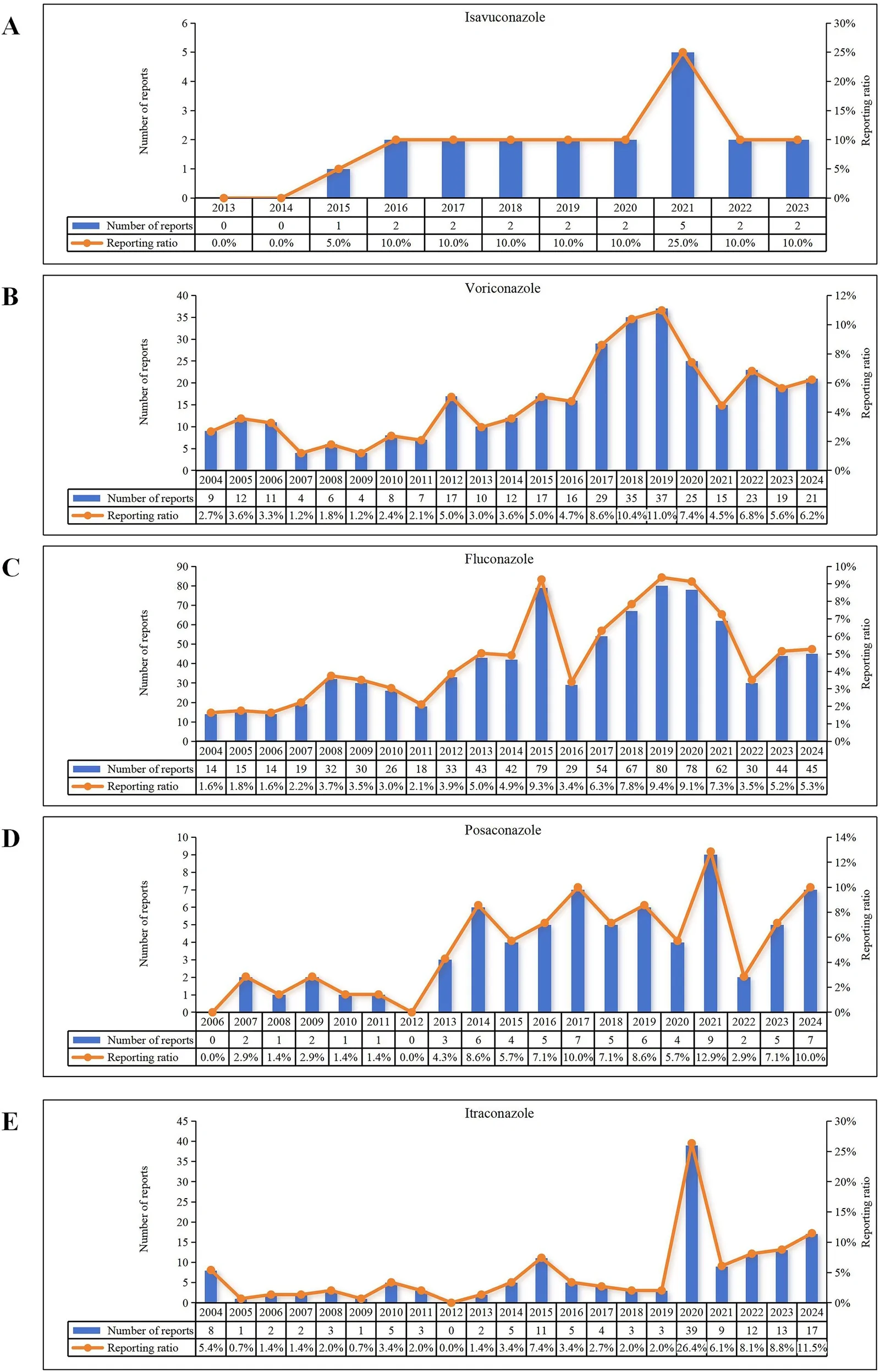
Yearly distribution of SCAR reports linked to Isavuconazole (A), Voriconazole (B), Fluconazole (C), Posaconazole (D), and Itraconazole (E).

The annual number and trends of SCARs cases associated with TADs were illustrated in Figure 2. The number of SCAR cases linked to Isavuconazole peaked in 2022 (5 cases) and then gradually declined. Voriconazole exhibited the highest number of SCAR cases among all TADs, peaking in 2019 (37 cases). Fluconazole showed a year-on-year increase from 2004, also hitting its peak in 2019 (80 cases). Posaconazole saw its highest count of SCAR cases in 2021 (9 cases), whereas Itraconazole hit its peak in 2020 (39 cases), and then showed a subsequent downward trajectory. Overall, a gradual increase in the number of SCAR cases linked to TADs is evident across the study period (Fig. 2).

### SCARs signal mining results for TADs

Four algorithms were used to examine the associations between TADs and SCARs. The criteria for positive signals were listed in Supplementary Table 2. In the analysis of associations between individual drugs and SCARs using multiple metrics, distinct findings emerged for each drug, along with an overall assessment for the TAD class (Table 2). Fluconazole demonstrated the strongest and most consistent safety signal. All four algorithms identified fluconazole as a positive signal, indicating a clear association with SCARs. Itraconazole also showed a consistent positive signal across all algorithms (ROR = 2.34, PRR = 2.32; EBGM05 = 2.05; IC025 = 0.99). Similarly, the combined analysis of all five antifungals yielded positive signals across all methods. Voriconazole presented mixed results with positive signals of the ROR algorithm (ROR = 1.62) and BCPNN (IC025 = 0.54), but with negative signals of PRR and EBGM. In contrast, posaconazole and isavuconazole did not generate positive signals by any of the four methods in this dataset. Overall, the analysis reveals a spectrum of statistical signal strengths among the antifungal agents studied, with fluconazole presenting the strongest and most consistent disproportionality signal, followed by itraconazole, whereas posaconazole and isavuconazole did not generate positive signals by any of the four methods in this dataset. This pattern should be interpreted as reflecting reporting associations rather than comparative clinical risk.

**Table 2.** Signal detection of Triazole antifungal drug-related SCARs.

Drugs	Number of reports	ROR (95%Cl)	PRR (χ^2^)	EBGM (EBGM05)	BCPNN (IC025)
Isavuconazole	20	0.61 (0.39–0.94)	0.61 (5.12)	0.61 (0.42)	−0.72 (−1.35)
Voriconazole	337	1.62 (1.46–1.79)	1.61 (87.74)	1.61 (1.48)	0.69 (0.54)
Fluconazole	854	5.2 (4.89–5.54)	5.05 (3336.82)	5.04 (4.79)	2.33 (2.24)
Posaconazole	70	1.23 (0.98–1.54)	1.22 (3.13)	1.22 (1.01)	0.29 (−0.04)
Itraconazole	148	2.34 (2.02–2.72)	2.32 (132.24)	2.32 (2.05)	1.21 (0.99)
Triazole antifungal drugs	1429	2.79 (2.66–2.93)	2.75 (1871.99)	2.75 (2.64)	1.46 (1.39)

Abbreviations: ROR, Reporting Odds Ratio; PRR, Proportional Reporting Ratio; BCPNN, Bayesian Confidence Propagation Neural Network; CI, confidence interval; EBGM, Empirical Bayes Geometric Mean; IC, Information Component.

### Prognostic analysis

**Figure 3.**
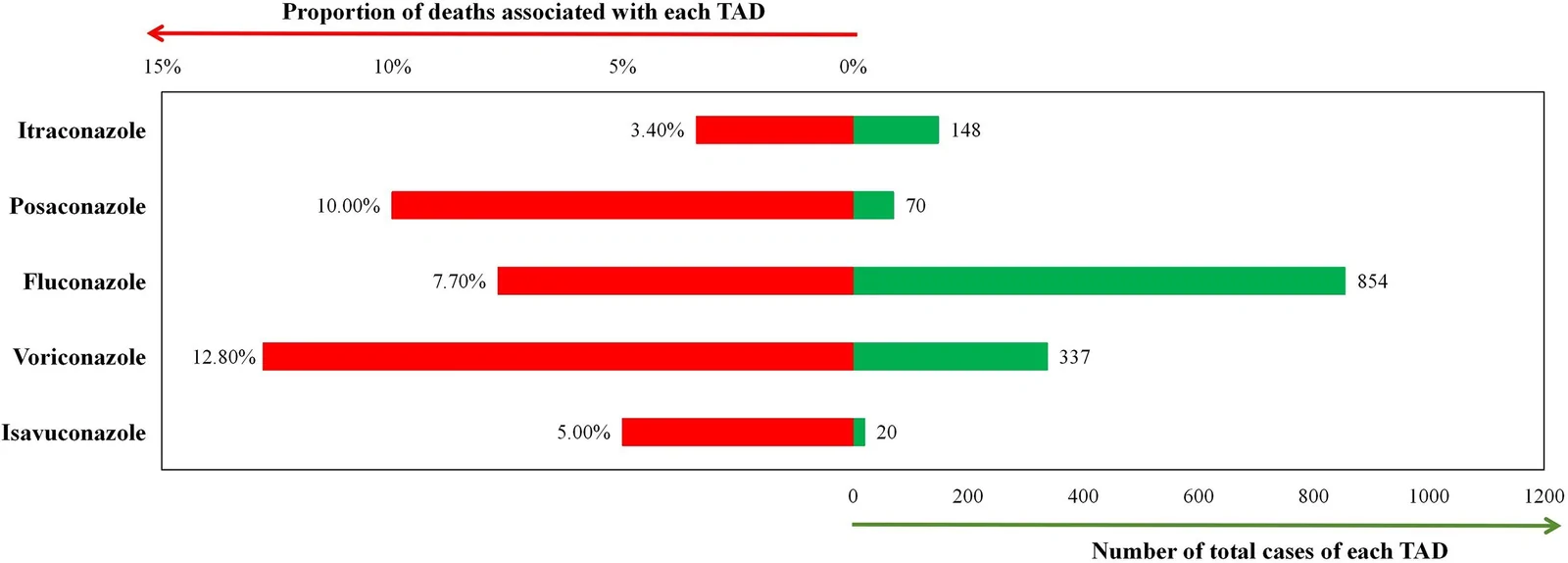
The proportion of SCARs deaths related to each TAD.

Treatment outcomes, specifically mortality rates following TAD therapy, were also compiled. Data for five drugs with distinct colors were used to differentiate between percentage and number (Fig. 3). Notable differences were observed in both the absolute number of reported cases and the associated mortality proportions. It is important to note, however, that these mortality proportions reflect unadjusted crude rates derived from spontaneous reports. Voriconazole, with the second-highest case count (*n* = 337), demonstrated the highest mortality proportion at 12.80%. Posaconazole, despite a considerably lower number of reported cases (*n* = 70), was associated with a notable mortality proportion of 10.00%. Isavuconazole, with the fewest reported cases (*n* = 20), showed a mortality proportion of 5.00%, while itraconazole (*n* = 148) exhibited the lowest mortality proportion at 3.40%.These findings suggest a dissociation between the frequency of SCAR reports and the severity of outcomes reflected by mortality. Voriconazole and posaconazole appear to be associated with more fatal outcomes in this descriptive analysis despite lower reporting frequencies, whereas fluconazole, though most commonly reported, shows a comparatively lower case-fatality proportion. However, as cautioned above, mortality differences observed in this spontaneous reporting database may largely reflect the severity of the underlying fungal disease, host immune status, and other clinical factors rather than direct drug effects. For example, voriconazole is often used in critically ill patients with invasive aspergillosis, a population inherently at higher baseline mortality risk. Itraconazole demonstrates both a lower frequency and the lowest mortality among the class. The results highlight the importance of considering both incidence and outcome severity in the pharmacovigilance profiling of antifungal agents, particularly for drugs like voriconazole.

### TTO analysis

**Figure 4.**
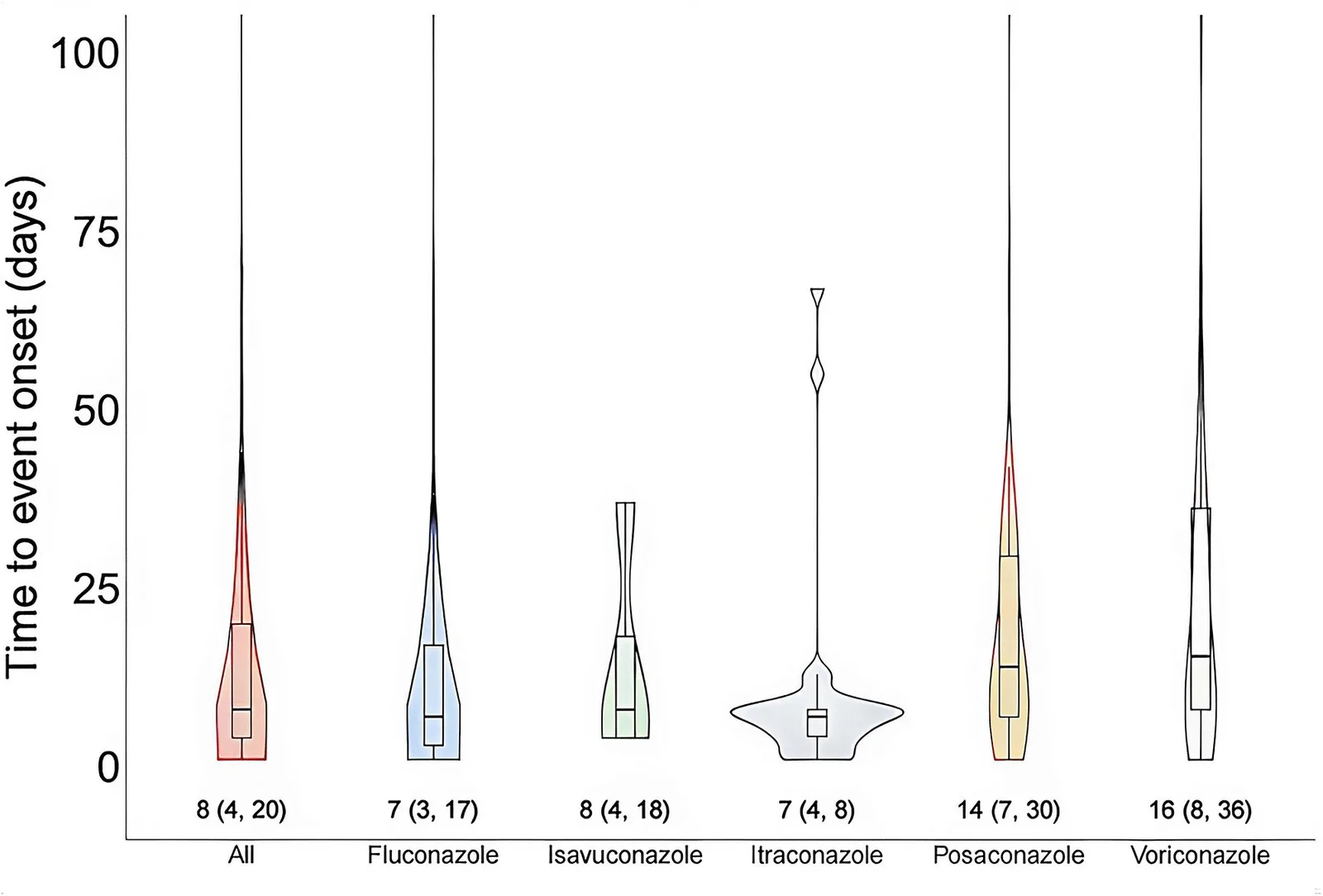
Temporal distribution of TADs-induced SCARs (time-to-onset, days).

TTO, the interval between drug exposure and AE occurrence, is a pivotal metric in drug safety surveillance. Weibull distribution curves were fitted to the time-to-onset data to generate smoothed density overlays on the histograms (Fig. 4), providing a visual characterization of the temporal distribution patterns for each drug. It is important to note that these curves were used solely for descriptive visualization of the observed TTO distributions; formal Weibull parameter estimation and hazard function interpretation were not performed, as the primary objective of this analysis was descriptive visualization rather than predictive modeling. Categorical variables were presented in the form of counts (percentages), while continuous variables were expressed as medians [interquartile ranges (IQR)].^.27^

Regarding the temporal distribution of SCARs induced by TADs, the median TTO was 8 days, with an IQR of 4–20 days. Among the different drug groups, patients treated with Voriconazole exhibited the longest TTO, averaging 16 days (IQR: 8–36 days), followed by those in the Posaconazole group (14 days, IQR: 7–30 days), Isavuconazole group (8 days, IQR: 4–18 days), Fluconazole group (7 days, IQR: 3–17 days), and Itraconazole group (7 days, IQR: 4–8 days). Specifically, the detailed temporal distribution for each TAD was illustrated in Figure 4. This pattern suggests that while the majority of SCARs occur early in the course of therapy, a clinically meaningful proportion may present after prolonged exposure, particularly for voriconazole and posaconazole. These descriptive observations should be interpreted with caution given the substantial missing data for EVENT_DT and START_DT in spontaneous reports.

### Heatmap of TADs and SCARs at the PT level

**Figure 5.**
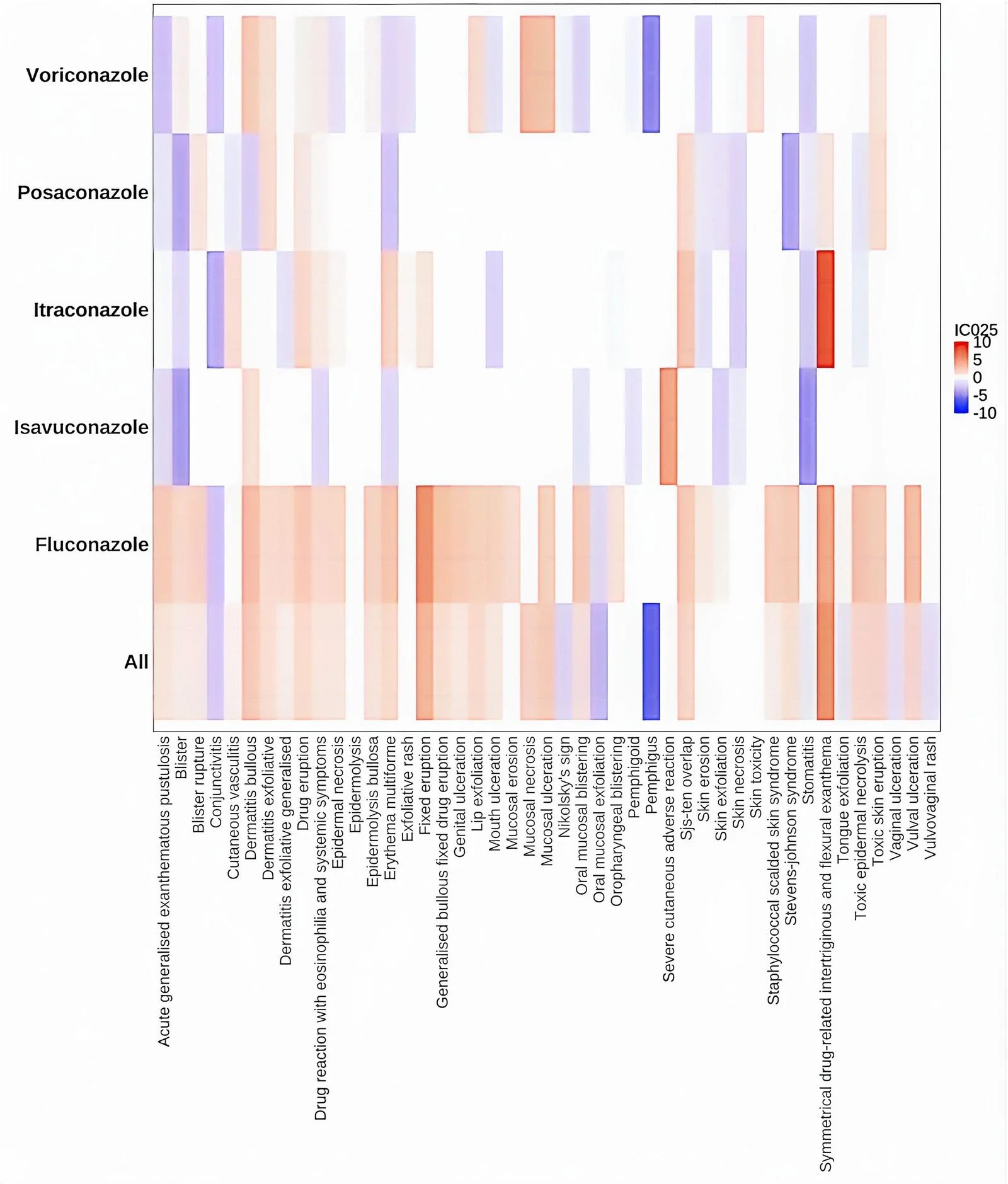
Heatmap of TAD and SCARs at the PT level.

A total of 45 signals (positive and negative) at the PT level across all five TADs were detected using the BCPNN method, as illustrated in Figure 5. Among these, the Fluconazole group exhibited the highest number of PT signals, with 40 cases (IC025 range: 0.06–5.68). This was followed by the Voriconazole group with 30 cases (IC025 range: 0.33–3.56), the Itraconazole group with 24 cases (IC025 range: 0.16–8.06), the Posaconazole group with 20 cases (IC025 range: 0.3–1.76), and the Isavuconazole group with 12 cases (IC025 range: 1.52–4.66).

Fluconazole showed the most significant associations with fixed eruption (IC025: 5.68) and symmetrical drug-related intertriginous and flexural exanthema (IC025: 4.56). Severe cutaneous adverse reaction (IC025: 4.66) demonstrated the strongest signal for Isavuconazole. Voriconazole exhibited notable associations with mucosal necrosis (IC025: 3.56) and mucosal ulceration (IC025: 3.23). SJS-TEN overlap (IC025: 1.76) revealed the strongest signal for Posaconazole. Within the Itraconazole group, symmetrical drug-related intertriginous and flexural exanthema (IC025: 8.06) showed the strongest association.

### Signals identified by the four algorithms

**Figure 6.**
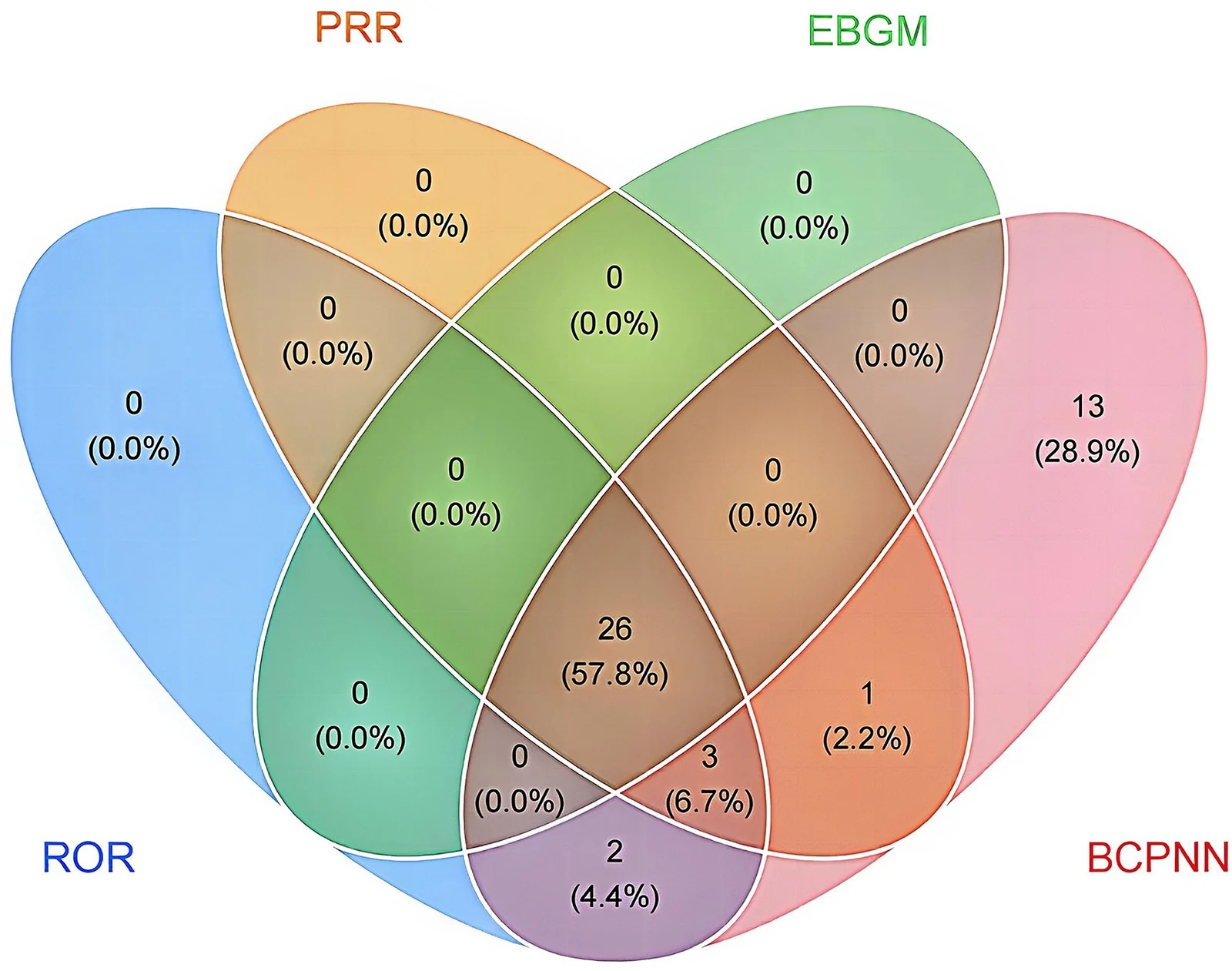
Signals identified by the four algorithms.

Among the analysis, a total of 26 PTs (57.8%) were simultaneously detected as positive signals by all four methods (Fig. 6), indicating that these AEs exhibit strong associations across multiple algorithms. Additionally, 13 PTs (28.9%) were exclusively detected by BCPNN and were not identified by the other methods, suggesting that BCPNN may be more sensitive to certain specific signals.

### Volcano plot of TAD-associated SCARs at the PT levels

**Figure 7.**
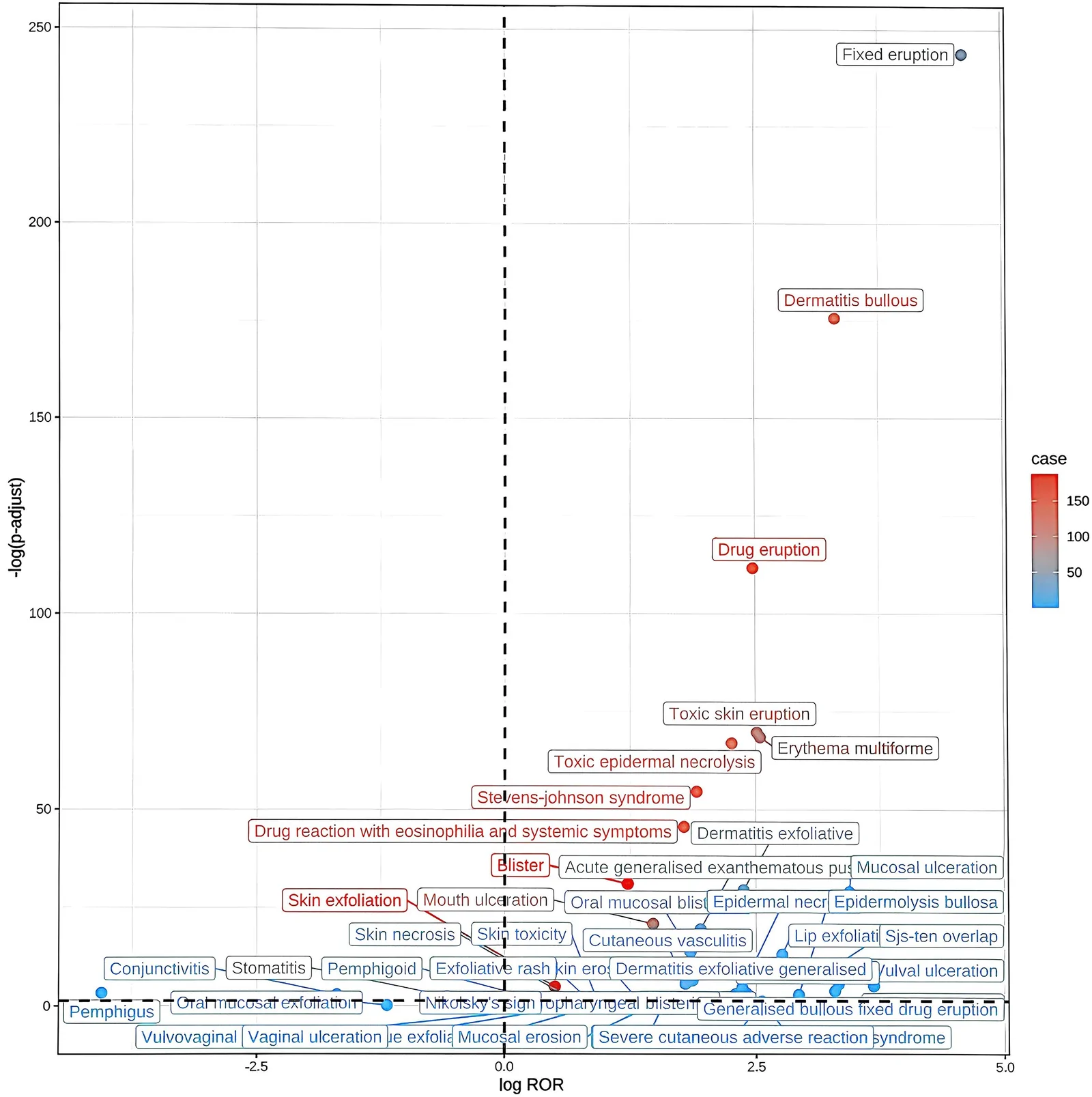
Volcano plot of TAD-induced SCARs at the PT levels.

The volcano plot, derived from disproportionality analysis of FAERS data, delineates the strength and statistical significance of associations between antifungal drug exposure and various SCARs (Fig. 7). The *x*-axis represents the magnitude of the drug-event association, while the *y*-axis depicts the negative logarithm of the adjusted *P*-value, reflecting the statistical confidence of each signal. Several high-signal and highly significant SCAR terms were prominently positioned in the upper-right quadrant of the plot. Notably, fixed eruption exhibited the strongest association (ROR = 24.43) with exceptional statistical significance (adjusted *P* = 1.54 × 10−^244^), representing the most robust safety signal identified. It is important to note, however, that while fixed drug eruption showed the strongest statistical association, it is generally considered less life-threatening than SJS/TEN; the high ROR reflects the frequency of reporting for this specific phenotype rather than clinical severity. Nonetheless, the signal warrants clinical awareness given the significant patient discomfort and potential for generalized bullous variants. Dermatitis bullous (ROR = 9.96), mucosal ulceration (ROR = 10.86), and vulval ulceration (ROR = 10.06) also demonstrated strong and highly significant signals, indicating substantial risk elevations for these specific manifestations. Established severe conditions such as drug eruption (ROR = 5.58), Stevens-Johnson syndrome (ROR = 3.76), Toxic epidermal necrolysis (ROR = 4.80), and drug reaction with eosinophilia and systemic symptoms (ROR = 3.44) were consistently positioned with high statistical significance, reinforcing their known association with antifungal therapy. Moderate yet significant signals were observed for erythema multiforme (ROR = 5.85), toxic skin eruption (ROR = 5.72), acute generalized exanthematous pustulosis (ROR = 3.85), and exfoliative dermatitis (ROR = 5.19), all with reproducible associations beyond chance.

Interestingly, several terms appear in the left quadrant, suggesting potential protective or lower-risk associations, though with varying statistical support. Pemphigus shows a markedly lower ROR (0.06) with moderate significance (*P* = 0.0003), while stomatitis (ROR = 0.67) and conjunctivitis (ROR = 0.31) also demonstrate negative associations with weaker statistical evidence. The volcano plot effectively stratifies SCAR terms into distinct risk categories: (1) high-impact, highly significant events (upper-right) warranting urgent clinical attention; (2) moderate but well-substantiated signals (middle-right) requiring vigilant monitoring; (3) lower-frequency events with borderline significance; and (4) potential negative associations (left side) that may reflect differential risk profiles or reporting artifacts. This comprehensive visualization underscores the spectrum of cutaneous toxicity associated with antifungal agents, highlighting both classic severe reactions and identifying potentially under-recognized high-risk manifestations such as fixed eruption and mucosal ulcerative conditions. The analytical approach enables prioritization of pharmacovigilance efforts based on both effect size and statistical reliability, supporting targeted risk minimization strategies in clinical practice.

## Discussion

The literature indicates that AEs are not uncommon among hospitalized patients, accounting for approximately 7% of all hospital admissions. Notably, cutaneous involvement occurs in up to 30%–45% of these AEs, among which about 2% may progress to severe forms, with mortality rates potentially reaching 10%–30% in such serious cases.^.28^ The widespread clinical use of TADs comes primarily from their broad-spectrum activity against a variety of fungal pathogens. However, there are many undiscovered and unreported adverse events as well as a lack of comparisons among different drugs. A study analyzing 952 SCAR reports of antifungal agents highlighted the high cutaneous risk associated with the 18–64 age group and extended observations to non-azole antifungals.^.29^ Another analysis of AE reports emphasized significant non–cutaneous signals including cholestasis, drug–induced liver injury, QT–prolongation, and renal impairment, with approximately 50% of cases resulting in severe outcomes.^30^ Further supporting this, an investigation of triazole–related records identified particularly strong signals in system organ classes such as endocrine disorders, metabolism and nutrition disorders, and skin disorders, alongside specific important medical events like respiratory failure, abnormal hepatic function, and hypokalemia.^.31^

In contrast to earlier reports, the present study provides a focused, granular, and clinically nuanced analysis of SCARs specifically associated with five TADs. Our work differs from prior literature in several key aspects. First, while previous studies reported SCARs as a collective entity or within broader organ classes, we dissected SCARs into precise PTs, such as fixed eruption, dermatitis bullous, Stevens–Johnson syndrome, toxic epidermal necrolysis, and mucosal ulceration, allowing for a detailed mapping of the dermatologic risk spectrum. Second, we employed a multi–algorithm disproportionality framework (ROR, PRR, EBGM, and BCPNN) to enhance signal robustness, rather than relying solely on ROR. This approach revealed important discrepancies: for example, voriconazole was flagged by ROR and BCPNN but not by PRR or EBGM, underscoring the value of convergent signal detection. Third, beyond signal strength, we integrated outcome–severity metrics by calculating mortality proportions for each drug, revealing a dissociation between reporting frequency and fatality risk, for instance, voriconazole showed a higher death proportion (12.8%) than fluconazole (7.7%) despite a lower overall SCAR signal. Finally, our volcano–plot visualization synthesized effect size and statistical significance to prioritize high–impact PTs, identifying fixed eruption as the most prominent signal, a finding not highlighted in earlier analyses.

We acknowledge an important limitation inherent to all pharmacovigilance studies using spontaneous reporting systems: the five TADs compared in this analysis have substantially different levels of market penetration, clinical usage volume, and approval timelines. For instance, fluconazole and itraconazole have been available for decades and are widely prescribed globally, whereas isavuconazole received regulatory approval more recently and is used in more restricted patient populations. Consequently, the raw number of SCAR reports, highest for fluconazole, may partly reflect differential prescribing frequency rather than differential intrinsic toxicity. More importantly, the strong disproportionality signal observed for fluconazole (ROR = 5.2) should not be equated with the highest intrinsic risk, as this signal is generated from reporting frequencies that are inherently influenced by the drug’s market share. Without access to denominator data on total patient exposure (e.g., defined daily doses per 1000 inhabitants per day or total prescription counts), it is not possible to determine whether fluconazole’s strong signal reflects true elevated risk or is primarily driven by its widespread use. However, our primary conclusions are based on disproportionality and Bayesian signal detection algorithms (ROR, PRR, BCPNN, EBGM), which compare the observed proportion of SCAR reports for each drug against the background proportion for all other drugs in the FAERS database. These methods inherently adjust for overall reporting volumes and thus mitigate the bias introduced by unequal drug utilization. Nevertheless, residual confounding due to unmeasured differences in treatment indications, patient populations, and reporting practices cannot be fully excluded. We therefore emphasize that our findings should be interpreted as hypothesis-generating signals of reporting associations, not as comparative measures of clinical risk. Future studies incorporating drug-exposure data (e.g., defined daily doses or prescription volumes) would help further validate the comparative safety signals observed here.

To align our findings with clinical practice, we compared pharmacovigilance signals against TAD indications per IDSA guidelines. Fluconazole showed the strongest and most consistent SCAR signal across all algorithms, likely influenced by its widespread use despite disproportionality adjustment. Voriconazole had the longest TTO (16 days) and highest mortality (12.8%), which may reflect both its dermatologic risk and the severe underlying conditions of treated patients. Our analysis further identified fixed eruption (fluconazole) and mucosal ulceration (voriconazole) as notable signals, extending prior case-report literature. Collectively, these results indicate that signal strength, onset timing, and outcome severity should be interpreted in combination when evaluating TAD cutaneous safety.

To enhance clinical applicability, we summarize below the key implications derived from our findings for each TAD: (1) Fluconazole: Exhibited the strongest and most consistent SCAR statistical signal across all four algorithms, with the highest absolute number of reports and a median TTO of 7 days. Clinicians should maintain a low threshold for evaluating cutaneous eruptions during the first week of therapy, particularly for fixed eruption and symmetrical drug-related intertriginous and flexural exanthema, which were the most prominently associated PTs. Despite the high reporting frequency, the observed mortality proportion (7.7%) was intermediate among the class. (2) Itraconazole: Showed consistent positive signals across all algorithms with a median TTO of 7 days and the lowest mortality proportion (3.4%) among the TADs. While the signal is robust, the favorable outcome profile in this dataset may offer some reassurance, though vigilance for SCARs remains warranted. (3) Voriconazole: Demonstrated mixed signal detection (positive by ROR and BCPNN, negative by PRR and EBGM) and the longest median TTO (16 days), with the highest crude mortality proportion (12.8%). The extended onset window suggests that SCAR monitoring should not be limited to the initial treatment phase but should extend well into the second and third weeks of therapy. Particular attention should be paid to mucosal ulceration and necrosis, which were prominent PT-level signals. The elevated mortality proportion, however, likely reflects the severity of underlying disease (invasive aspergillosis in immunocompromised hosts) rather than drug-specific fatal toxicity. (4) Posaconazole: Did not generate positive signals by any algorithm in this dataset, with a median TTO of 14 days and a mortality proportion of 10%. The absence of a statistical signal, coupled with the relatively low number of reports (*n* = 70), may reflect lower clinical utilization and/or a more favorable cutaneous safety profile, but the small sample size substantially limits statistical power and precludes definitive conclusions regarding its safety. Larger post-marketing studies are needed to confirm this apparent favorable profile. SJS-TEN overlap was the strongest PT-level association identified. (5) Isavuconazole: Also showed no positive signals by any algorithm, with the fewest reports (*n* = 20) and a mortality proportion of 5%. As the newest agent in this class, its limited reporting volume likely reflects restricted market penetration and usage in highly selected patient populations, resulting in low statistical power to detect any but the most pronounced signals. Therefore, the absence of positive signals should not be interpreted as evidence that isavuconazole is entirely ‘‘SCAR-safe.’’ Nonetheless, severe cutaneous adverse reaction was the strongest PT-level signal, warranting continued post-marketing surveillance.

A critical limitation of comparing mortality proportions across different triazole antifungals in this study is the strong potential for confounding by disease severity and patient population. Triazole antifungals are not interchangeable in clinical practice; they are used for different indications, in different patient populations, and at different stages of disease. For instance, voriconazole is a first-line agent for invasive aspergillosis, typically affecting severely immunocompromised patients with high baseline mortality, while fluconazole is widely used for less severe conditions such as mucosal candidiasis. Similarly, posaconazole is often employed as prophylaxis in patients with prolonged neutropenia or hematopoietic stem cell transplant recipients, populations with substantial intrinsic mortality risk. Therefore, the higher crude mortality proportions observed for voriconazole and posaconazole compared to fluconazole and itraconazole should not be interpreted as evidence of differential drug-induced fatal toxicity. Rather, these differences likely reflect, at least in part, the underlying risk profiles of the treated populations. In the absence of patient-level data on disease severity, comorbidities, and treatment indications, such comparisons remain hypothesis-generating. Future studies using propensity score matching or multivariable adjustment with electronic health record data are needed to disentangle drug effects from disease effects on mortality. In our analysis, fluconazole and itraconazole generated consistent positive signals across all four algorithms, whereas voriconazole yielded mixed results. Posaconazole and isavuconazole showed no significant signals in this dataset. These findings, while hypothesis-generating, may assist clinicians in recognizing differential reporting patterns among TADs.

Case reports of SJS following fluconazole^32^ and TEN during voriconazole treatment^33–36^ have previously drawn attention to the potential for TADs to induce severe mucocutaneous reactions. Currently, our research indicated that Fluconazole dominated in terms of SCARs reports. Across all years, the number of SCARs associated with Fluconazole far surpassed that of the other four drugs, displaying the most pronounced trend line. Notably, an abnormal peak occurred between 2014 and 2016, making Fluconazole the primary source of monitoring signals. This suggests that within this surveillance system, Fluconazole had the most abundant safety data or, conversely, the highest number of reported potential safety issues. Voriconazole ranked second, with its report count consistently holding the second position. Similar to Fluconazole, it demonstrated sustained attention. Posaconazole had a relatively low number of reports, with a stable trend. Isavuconazole, being a newer drug on the market, had the lowest report volume, with its curve consistently at the bottom, significantly lower than those of other drugs. This may be attributed to its later market introduction and relatively smaller clinical usage volume.

The clinical and financial burden of SCARs is substantial. In particular, the mortality rate linked to drug-induced SJS/TEN had been documented to soar as high as 50%.^.37^ In this study, Voriconazole (12.8%) and Posaconazole (10%) exhibited the highest mortality rates among the TADs, while Fluconazole, Isavuconazole, and Itraconazole had the lowest mortality rates. Additionally, the median TTO of SCARs following TAD administration was 8 days. Among different TAD groups, Voriconazole (16 days) had the longest TTO, followed by Posaconazole, Isavuconazole, Fluconazole, and Itraconazole. These findings underscore significant differences in the timing of SCARs onset across various TADs, which can assist clinicians in predicting and monitoring these potentially severe adverse reactions.

At the PT level, 45 distinct signals were identified across the TADs, with fluconazole showing the highest number of PT associations. Notable drug-specific associations included fixed eruption and symmetrical drug-related intertriginous and flexural exanthema with fluconazole; mucosal necrosis and mucosal ulceration with voriconazole; SJS-TEN overlap with posaconazole; and severe cutaneous adverse reaction with isavuconazole. Consistent with our inclusion criteria, fixed drug eruption was included as a SCAR in this analysis; however, we acknowledge that its clinical severity is generally lower than that of SJS/TEN, and the strong signal observed primarily reflects reporting frequency rather than life-threatening risk. Itraconazole was most strongly associated with symmetrical drug-related intertriginous and flexural exanthema. These PT-level findings highlight distinct phenotypic patterns that may inform targeted monitoring during TAD therapy.

These data can assist clinicians in weighing the efficacy and potential risks of TADs when selecting a drug. Additionally, this study analyzed the distribution of different PTs in a volcano plot. By examining the volcano plot of PTs within SCARs induced by TAD treatment, generated using ROR method, we gained a comprehensive understanding of the association strength and statistical significance between different PTs and TADs. The results provide clinicians with evidence-based guidance for prescribing TADs, facilitating early identification of high-risk SCARs and informing targeted preventive and monitoring strategies. Through a systematic retrospective analysis of the FAERS database, this study also explored the time intervals between drug initiation and SCAR onset and identified which TADs demonstrated stronger safety signals for specific clinical phenotypes. These insights are instrumental in supporting early clinical intervention, improving risk stratification, and promoting timely and tailored patient management, thereby enhancing the safety profile of TADs in real-world clinical settings.

This study has several important limitations inherent to the FAERS database and spontaneous reporting systems that should be carefully considered when interpreting the findings. First, the FAERS database lacks denominator data on the total number of patients exposed to each drug, as well as information on treatment duration and dosage. Consequently, we cannot calculate true incidence rates (e.g., number of SCAR cases per 10 000 patient-years) or compare absolute risks across triazole antifungals. Our disproportionality analyses (ROR, PRR, BCPNN, EBGM) compare observed versus expected reporting proportions, which mitigate but do not eliminate the bias introduced by differential drug utilization. Second, spontaneous reporting systems are subject to multiple forms of reporting bias, including under-reporting, selective reporting (more severe or unexpected events are more likely to be reported), and stimulated reporting (media attention or regulatory actions may temporarily increase reporting for specific drugs). These biases may differentially affect the five triazole antifungals depending on their market age, clinical awareness, and regulatory history, potentially distorting comparative signal strengths. Third, confounding by indication and disease severity is a major concern. Triazole antifungals are used in different patient populations and for different clinical indications. Patients receiving voriconazole often have invasive aspergillosis with critical illness, whereas those receiving fluconazole may have less severe conditions. The higher crude mortality proportion observed for voriconazole almost certainly reflects, at least in part, the poorer underlying prognosis of these patients rather than a direct drug effect. Our disproportionality analyses adjust for background reporting rates but cannot fully account for such indication-based confounding due to the absence of detailed clinical data.

Fourth, this study did not perform formal causality assessment for individual reports. The algorithms used detect statistical associations, not causal relationships. A positive signal indicates that SCARs are reported more frequently for a given drug than for others in the database, but it does not prove that the drug caused the event in any specific case. Causality assessment would require individual case review applying standardized instruments such as the Naranjo algorithm or WHO-UMC criteria, which is not feasible in a large-scale database study. Fifth, the FAERS database contains limited information on clinically relevant confounding factors, including renal and hepatic function, concomitant medications (beyond the reported suspect drugs), comorbidities, smoking status, alcohol use, and genetic predispositions. The absence of these variables precludes multivariable adjustment and may lead to residual confounding. Sixth, the small number of reports for posaconazole (*N* = 70) and isavuconazole (*N* = 20), coupled with the latter’s recent regulatory approval (2015), substantially limits the statistical power to detect signals for these agents. Consequently, the absence of positive signals for these drugs should not be interpreted as evidence of no risk or as definitive proof of superior safety; rather, it reflects the current state of reporting and underscores the need for continued pharmacovigilance as clinical exposure accumulates. Additional limitations includethe incomplete data fields (e.g., TTO was missing for many reports), limiting generalizability to other regions. While Weibull-smoothed density curves were used to visualize the temporal distribution of TTO, formal Weibull parameter estimation and hazard pattern interpretation were beyond the scope of this descriptive analysis. The substantial proportion of reports with missing or incomplete dates for EVENT_DT and START_DT further limits the reliability of TTO calculations and precludes robust time-to-event modeling. Future studies with complete longitudinal medication data and time-to-event analytic frameworks are needed to rigorously characterize the hazard patterns of TAD-induced SCARs. Finally, the precise molecular mechanisms by which TADs induce SCARs remain incompletely understood, which limits our ability to interpret the observed associations in a pathophysiological context. Despite these limitations, the FAERS database remains a valuable resource for hypothesis generation and post-marketing safety surveillance. Our findings provide signal detection that can inform clinical vigilance and guide future confirmatory studies using more rigorous epidemiological designs, such as cohort studies with exposure data or nested case-control studies with detailed clinical covariates.

Based on the current evidence and identified limitations, several avenues for further investigation warrant consideration. First, pharmacists can play a valuable role in monitoring drug-induced AEs and educating patients.^38–41^ Thus, the participation of clinical pharmacists in the treatment team may contribute to patient safety management. Second, pharmacogenomics (e.g., drug-metabolizing enzymes), *in vitro* skin models, and single-cell sequencing could be combined to explore the molecular mechanisms by which TADs induce severe cutaneous AEs. In particular, future studies should investigate HLA associations with TAD-induced SCARs, analogous to established pharmacogenomic biomarkers such as HLA-B58:01 for allopurinol-induced SJS/TEN and HLA-B15:02 for carbamazepine-induced SJS/TEN. Given the immunopathological basis of SCARs, identifying specific HLA risk alleles for individual triazoles could enable pre-emptive screening and guide antifungal selection in high-risk populations. Third, integrating electronic health records, genomic databases, and cross-regional pharmacovigilance data could help build more comprehensive risk prediction models. Fourth, longitudinal medication data and formal time-to-event analysis (e.g., Cox proportional hazards models with complete exposure data) could more accurately characterize the temporal relationship from drug initiation to SCAR onset and identify risk factors associated with early versus late onset. Fifth, network pharmacology and systems toxicology methods could explore the ‘class effects’ and heterogeneous reaction patterns of antifungal drugs. Sixth, a SCAR risk stratification tool based on risk factors such as age, liver and kidney function, and comorbidities should be developed, along with differentiated monitoring plans for different risk levels. Finally, efforts should be made to establish a structured AE reporting template, strengthen the collection of key clinical details such as rash morphology, pathological findings, and drug discontinuation outcomes, and enhance the reliability and research usability of spontaneously reported data.

## Conclusion

This research identified and quantified statistical associations for SCARs among triazole antifungals using a spontaneous reporting database. Fluconazole and itraconazole exhibited the strongest SCAR statistical signals among TADs in this dataset, warranting heightened clinical vigilance. The absence of signals for posaconazole and isavuconazole should be interpreted with caution due to limited reporting volumes and statistical power. These agents therefore appear to have a more favorable cutaneous safety profile in this dataset, though larger post-marketing cohorts and longer observation periods are needed to confirm these preliminary findings. It must be emphasized, however, that these signals reflect reporting associations and may be influenced by differential drug utilization; fluconazole’s strong signal should be interpreted in the context of its widespread global use, and the absence of signals for newer agents does not constitute proof of superior safety. The significant variation in TTO and mortality across TADs underscores the need for drug-specific monitoring strategies, although mortality differences should be interpreted with caution given the strong potential for confounding by disease severity and patient population. These findings support mechanism exploration, early risk recognition, and informed antifungal selection when interpreted in the context of the aforementioned limitations. The findings should be interpreted with caution given the inherent limitations of spontaneous reporting data, including the absence of exposure denominators, potential confounding by indication, and lack of formal causality assessment. Confirmatory studies using exposure-adjusted designs and formal causality assessment are needed to validate the observed signals.

## Supplementary Material

myag084_Supplemental_File
