# Supplementary material for: A retrospective study comparing the severe cutaneous adverse reactions in patients receiving triazole antifungal therapy and literature analysis
Source: Med Mycol. 2026 Aug 17;64(8):myag084. doi: 10.1093/mmy/myag084 (PMC13502895; doi:10.1093/mmy/myag084)
Supplement: Supplementary file 1 [file myag084_supplemental_file.docx]

**Supplementary Table 1.** Four grid table of the proportional imbalance methods.

| **Drug class** | **Number of reports of the target adverse events** | **Number of reports of the other adverse events** | **Total** |
| --- | --- | --- | --- |
| Suspect drug | a | b | a+b |
| Other drugs | c | d | c+d |
| Total | a+c | b+d | N=a+b+c+d |

Note: a: the number of reports containing both the suspect drug and the suspect adverse events; b: the number of reports containing the other adverse events with suspect drug; c: the number of reports containing the other drugs with the target adverse events; d: the number of reports containing other drugs and other adverse events; N: the number of all reports.

**Supplementary Table 2.** Formulas and thresholds of the four algorithms.

| **Algorithms** | **Equation** | **Criteria** |
| --- | --- | --- |
| ROR |  | a ≥ 3，95%CI（low limit）＞ 1 |
| PRR |  | a ≥ 3，PRR ≥ 2，χ^2^ ≥ 4 |
| BCPNN |  | IC025 ＞ 0 |
| EBGM |  | EBGM05 ＞ 2 |

Note: a: the number of reports containing both the suspect drug and the suspect adverse events; b: the number of reports containing the other adverse events with suspect drug; c: the number of reports containing the other drugs with the target adverse events; d: the number of reports containing other drugs and other adverse events; N: the number of all reports. Abbreviations: ROR, Reporting Odds Ratio; PRR, Proportional Reporting Ratio; BCPNN, Bayesian Confidence Propagation Neural Network; CI, confidence interval; EBGM, Empirical Bayes Geometric Mean; IC, Information Component.
